# Metagenomics reveals the response of desert steppe microbial communities and carbon-nitrogen cycling functional genes to nitrogen deposition

**DOI:** 10.3389/fmicb.2024.1369196

**Published:** 2024-03-26

**Authors:** He Ye, Yu Zhao, Shilong He, Zhendan Wu, Mei Yue, Mei Hong

**Affiliations:** ^1^Inner Mongolia Key Laboratory of Soil Quality and Nutrient Resources, College of Grassland, Resources and Environment, Inner Mongolia Agricultural University, Hohhot, China; ^2^Key Laboratory of Agricultural Ecological Security and Green Development at Universities of Inner Mongolia Autonomous Region, Hohhot, China

**Keywords:** metagenomics, soil microbiome, carbon cycle function, nitrogen cycle function, nitrogen deposition

## Abstract

**Introduction:**

Nitrogen (N) deposition seriously affects the function of carbon (C) and N cycling in terrestrial ecosystems by altering soil microbial communities, especially in desert steppe ecosystems. However, there is a need for a comprehensive understanding of how microorganisms involved in each C and N cycle process respond to N deposition.

**Methods:**

In this study, shotgun metagenome sequencing was used to investigate variations in soil C and N cycling-related genes in the desert steppe in northern China after 6 years of the following N deposition: N0 (control); N30 (N addition 30 kg ha^−1^ year^−1^): N50 (N addition 50 kg ha^−1^ year^−1^).

**Results:**

N deposition significantly increased the relative abundance of *Actinobacteria* (*P* < 0.05) while significantly decreased the relative abundances of *Proteobacteria* and *Acidobacteria* (*P* < 0.05). This significantly impacted the microbial community composition in desert steppe soils. The annual addition or deposition of 50 kg ha^−1^ year^−1^ for up to 6 years did not affect the C cycle gene abundance but changed the C cycle-related microorganism community structure. The process of the N cycle in the desert steppe was affected by N deposition (50 kg ha^−1^ year^−1^), which increased the abundance of the *pmoA-amoA* gene related to nitrification and the *nirB* gene associated with assimilation nitrite reductase. There may be a niche overlap between microorganisms involved in the same C and N cycling processes.

**Discussion:**

This study provides new insights into the effects of N deposition on soil microbial communities and functions in desert steppe and a better understanding of the ecological consequences of anthropogenic N addition.

## 1 Introduction

Desert steppe is a xeric grassland ecosystem representing the transition from grassland to desert (Guo et al., 2022). It is an important grassland type in the grasslands of Europe and Asia, accounting for comprising ~11% of Inner Mongolia's steppe area (Qiu et al., 2022). The inner Mongolian desert steppe is not only an ecological barrier in northern China but also one of the world's most sensitive types of semi-arid and arid ecosystems (Gong et al., 2022). Nitrogen (N) deposition has been an important component in the global N cycle with increasing anthropogenic reactive N emissions (Galloway et al., 2008; Liu et al., 2011). According to reports, the N deposition in desert steppe has been ~4 kg ha^−1^ year^−1^ since the 1980s (Liu et al., 2011). However, recent observations indicate that N deposition in the region is estimated to be 15.00–17.00 kg ha^−1^ year^−1^ (Zhang, 2021; Li, 2022). Moreover, N deposition will be maintained for a long time in developing countries like China due to its rapid agricultural, industrial, and urban development (Yin et al., 2022). Determining the response of desert steppe soil microbial communities to N deposition is crucial to accurately assess changes in the processes of semi-arid and arid ecosystems and to develop the strategies for the effective mitigation and maintenance of ecosystem health under future global climate change scenarios.

Changes in soil microbial communities may play a crucial role in regulating primary productivity and nutrient cycling (Van Der Heijden et al., 2008; Philippot et al., 2024). The increase in N deposition can enhance soil available N (NH${\;}_{4}^{+}$-N and NO${\;}_{3}^{-}$-N) and alter soil pH (Khalili et al., 2016; Stevens et al., 2022). These consequences may generate bottom-up cascading effects, leading to alterations in the composition and functionality of soil microbial communities (Widdig et al., 2020; Frey et al., 2023). A global study suggests that N addition alters microbial community structure, possibly due to microbial adaptation to N excess (Wang et al., 2023a). It has also been reported that sustained increases in N deposition have significant adverse effects on soil microorganisms and their functioning, and that the adverse effects of N application on soil microbial abundance and composition increase with the amount and duration of N deposition (Compton et al., 2004; Zhang et al., 2018). However, desert steppe has long been subjected to N limitation, and further research is needed to investigate the impacts of N deposition on soil microbial composition and abundance (Zhang et al., 2023b).

Nitrogen addition typically enhances N cycling rates, such as gross and potential nitrification and potential denitrification, and can also alter the abundance of key microbial groups involved in N cycling, such as *Actinobacteria* and *Alphaproteobacteria*) (Barnard et al., 2006; Le Roux et al., 2016; Assémien et al., 2017). Microorganisms drive fundamental N transformation processes in various ecosystems, such as N fixation, N mineralization, nitrification, and denitrification (Geisseler et al., 2010; Jansson and Hofmockel, 2020). N-related functional genes, such as *pmoA-amoA, pmoB-amoB*, and *pmoC-amoC* genes for nitrification, *narG, nirK*, and *nosZ* genes for denitrification, and *narB* and *nirB* genes for assimilative N reduction, are responsible for the synthesis of key enzymes in nitrogen transformation (Kuypers et al., 2018; Silva et al., 2022; Fudjoe et al., 2023). A meta-analysis showed that N deposition significantly increased nitrification-related functional genes and did not affect substantially denitrification *narG* genes (Song and Niu, 2022). Another study showed that N deposition significantly reduced the abundance of the functional genes *nifH, a moA*, and *nirK*, leading to suppression of N fixation, nitrification, and denitrification (Zhang et al., 2023a). Atmospheric N deposition leads to N enrichment in soil, inducing changes in plant growth and soil biological activity, thereby affecting global carbon (C) and N cycling (Püspök et al., 2023). Nitrogen deposition affects soil C cycle functional genes more than N cycle functional genes (Li et al., 2022b; Hagh-Doust et al., 2023). It is reported that N deposition increased the relative abundance of genes related to labile C degradation, on the contrary, the relative abundance of functional genes related to degradation of more recalcitrant C did not change or decrease (Ma et al., 2022). Nitrogen deposition may also increase the metabolic capacity for soil C stabilization by promoting the colonization of fast-growing bacteria and stimulating functional genes associated with C-degrading activity (Ma et al., 2022).

Nitrogen deposition has inconsistent effects on soil microbial community composition and C-N functions in different ecosystems. However, there are still many uncertainties about how N deposition interacts with biotic and abiotic factors to affect soil microbial communities in a desert steppe (Yu et al., 2023). Therefore, this study employed metagenomic sequencing technology to assess the effects of N deposition on soil microbial communities in the desert steppe in northern China. Our main questions were: (1) How does N deposition affect the composition of soil microbial communities and the abundance of functional genes related to C–N cycling in desert steppe ecosystems? (2) How do microorganisms involved in the same C–N cycling processes respond to N deposition and, (3) What environmental factors are associated with changes in their relative abundance? Our research findings can contribute to assessing and predicting the response of soil microbial communities in arid ecosystems to changes in N deposition.

## 2 Materials and methods

### 2.1 Study site

The field experiment was established at Siziwang Banner (41°46′43.6″N, 111°53′41.7″E, with an elevation of 1,456 m), an arid region in Inner Mongolia, northern China. The average annual precipitation is 280 mm, with cumulative precipitation falling during the growing season from May to October, constituting ~70% of the total precipitation throughout the year. The annual average temperature is 3.4°C. The soil at the study site is a sandy loam texture classified as a Haplic Calcisol based on the United Nations Food and Agricultural Organization (FAO) soil classification system. The plant communities are dominated by *Stipa breviflora* Griseb., *Neopallasia pectinata* (Pall.) Poljak., *Artemisia scoparia* Waldst. et Kit., *Kochia prostrata* (L.) Schrad., and *Cleistogenes songorica* (Roshev.) Ohwi.

### 2.2 Experimental design

The simulated N deposition experiments were established at the desert steppe site in December 2015. Three N-addition treatments were applied: (i) the control treatment (N0, no N addition), (ii) the first N addition treatment (N30, 30 kg ha^−1^ year^−1^), and (iii) the second N addition treatment (N50, 50 kg ha^−1^ year^−1^). NH_4_NO_3_ was mixed with purified water and sprinkled evenly on each plot using a sprayer to simulate wet deposition to mirror the natural seasonal N deposition pattern from May to September. From October of the same year to April of the following year, NH_4_NO_3_ was mixed with soil and broadcasted evenly by hand to simulate dry deposition (Supplementary Table S1). N was applied once per month at the beginning of the month. The monthly N application rate was determined by the percentage of the average monthly precipitation in the last 5 years relative to total annual precipitation (0.51%, 1.17%, 1.13%, 2.82%, 6.36%, 18.25%, 27.32%, 13.04%, 15.33%, 6.91%, 5.79%, and 1.37% from January to December, respectively). These experiments were planned using a randomized block design with four replicate blocks. Each plot measured 7 × 7 m, and 1 m walkways separated the plots.

### 2.3 Plant, soil, and soil microbial sampling

During the peak growing season (Mid-August) of vegetation in 2021, two randomly selected 0.5 × 0.5 m sample areas were chosen from each N treatment (four repetitions, with two sample squares per repetition. Take the average of two sample squares for each repeat). The aboveground parts of all plants within the sample squares were collected. Simultaneously, a core drill (diameter = 7 cm) was used to collect soil samples from the 0–10 cm depth, and the plant roots were collected. After collecting samples from each plot, the sampling apparatus was sterilized with 75% ethanol to prevent sample contamination. The collected aboveground parts and roots of plants were dried at 65°C for 48 h to measure the aboveground and belowground biomass. Soil samples were collected using a core drill to a depth of 10 cm. After removing roots and stones and gently mixing the soil, each sample was placed into a sterile plastic bag. One subsample was air-dried, sieved (2 and 0.15 mm), and used to assess soil physicochemical properties; one subsample was stored at −20°C to determine ammonium N (NH${\;}_{4}^{+}$-N) and nitrate N (NO${\;}_{3}^{-}$-N) concentrations. One subsample was immediately stored at −80°C to shotgun metagenomic sequencing.

### 2.4 Shotgun metagenomic sequencing

The total genomic DNA of the soil samples was extracted using the Mag-Bind^®^ Soil DNA Kit (Omega Bio-tek, Norcross, GA, US) according to manufacturer's instructions. The concentration and purity of extracted DNA were determined with TBS-380 and NanoDrop2000, respectively. DNA extract quality was checked on 1% agarose gel. DNA extract was fragmented to an average size of about 400 bp using Covaris M220 (Gene Company Limited, China) for paired-end library construction. The paired-end library was constructed using NEXTFLEX Rapid DNA-Seq (Bioo Scientific, Austin, TX, USA). Adapters containing the full complement of sequencing primer hybridization sites were ligated to the blunt-end of fragments. Paired-end sequencing was performed on Illumina NovaSeq (Illumina Inc., San Diego, CA, USA) at Majorbio Bio-Pharm Technology Co., Ltd. (Shanghai, China) using NovaSeq 6000 S4 Reagent Kit v1.5 (300 cycles) according to the manufacturer's instructions (www.illumina.com). Raw reads were found in the National Center for Biotechnology Information (NCBI) Sequence Read Archive database (accession numbers: PRJNA979949).

The data were analyzed on the free online platform of Majorbio Cloud Platform (www.majorbio.com). Briefly, the paired-end Illumina reads were trimmed of adaptors, and low-quality reads (length < 50 bp or with a quality value < 20 or having N bases) were removed by fastp (Chen et al., 2018; https://github.com/OpenGene/fastp, version 0.20.0). These high-quality reads were then assembled to contigs using MEGAHIT (Li et al., 2015) (parameters: kmer_min = 47, kmer_max = 97, step = 10; https://github.com/voutcn/megahit, version 1.1.2), which makes use of succinct de Bruijn graphs. Contigs with a length ≥300 bp were selected as the final assembling result, and then the contigs were used for further gene prediction and annotation.

Open reading frames (ORFs) from each assembled contig were predicted using Prodigal (Hyatt et al., 2010)/MetaGene (Noguchi et al., 2006) (http://metagene.cb.k.u-tokyo.ac.jp/). The predicted ORFs with a length ≥100 bp were retrieved and converted into amino acid sequences using the NCBI translation table (http://www.ncbi.nlm.nih.gov/Taxonomy/taxonomyhome.html/index.cgi?chapter=tgencodes#SG1).

A non-redundant gene catalog was constructed using CD-HIT (Fu et al., 2012) (http://www.bioinformatics.org/cd-hit/, version 4.6.1) with 90% sequence identity and 90% coverage. High-quality reads were aligned to the non-redundant gene catalogs to calculate gene abundance with 95% identity using SOAPaligner (Li et al., 2008) (http://soap.genomics.org.cn/, version 2.21).

Representative sequences of the non-redundant gene catalog were aligned to the Non-Redundant Protein Sequence (NR) database with an *e*-value cutoff of 1e^−5^ using Diamond (Buchfink et al., 2015) (http://www.diamondsearch.org/index.php, version 0.8.35) for taxonomic annotations. The KEGG annotation was conducted using Diamond (Buchfink et al., 2015; http://www.diamondsearch.org/index.php, version 0.8.35) against the Kyoto Encyclopedia of Genes and Genomes database (http://www.genome.jp/keeg/) with an e-value cutoff of 1e^−5^.

The calculation method of species and gene abundance is Reads Per Kilobase Million (RPKM) (Lawson et al., 2017):


$$\begin{array}{l} RPKM_{i}=\frac{R_{i}\times 10^{6}}{L_{i}\times \overset{n}{\underset{1}{\sum }}\left(R_{j}\right)} \end{array}$$

Where *R*_*i*_ represents the abundance value of Gene_*i*_ in a given sample, i.e., the number of Reads compared to Gene_*i*_ in that sample; *L*_*i*_ means the nucleotide length of Gene_*i*_; and $\overset{n}{\underset{1}{\sum }}\left(R_{j}\right)$ represents the sum of reads corresponding to all genes in that sample.

### 2.5 Data analyses

Microsoft Excel 2019 and R-4.2.3 were used for the statistical data analyses. The β*-*diversity values of the soil microbial community and function were visualized with nonmetric multidimensional scaling (NMDS) plots based on the Bray–Curtis dissimilarity matrix. Permutational multivariate analysis of variance (PERMANOVA) was used to test the significance of changes in the soil microbial community under the N treatments. Differential relative abundance of KEGG Pathway (level-3) was detected by the Wilcoxon rank-sum test with an adjusted *P* < 0.05 (corrected by the Benjamini–Hochberg). Enrichment in controls or N treatment was determined according to the higher mean rank-sum. Pathways were considered significantly different if the |reporter score| > 1.65, corresponding to 95% confidence to a normal distribution. The Shapiro–Wilk test was used to assess normality before testing for significance. One-way ANOVA was employed to analyze the effects of N treatments on aboveground biomass (AGB), belowground biomass (BGB), soil pH, soil organic C (SOC), dissolved organic C (DOC), total N (TN), NO${\;}_{3}^{-}$-N and NH${\;}_{4}^{+}$-N content, C cycling genes (*cbbL, cbbS, accA, accB, accC, accD, bccA, acsA, nifj*), and N cycling associated genes (*pmoA-amoA, pmoB-amoB, pmoC-amoC, nosR, nosZ, nirB, nirK, norB, narB, narG, narH, P* < 0.05). Carbon cycle functional gene selection was based on the Calvin-Benson-Basshamcycle (CBB), tricarboxylic acid cycle (rTCA), Reductive acetyl-CoA pathway (Wood-Ljungdahl pathway), 3-Hydroxypropionate bicycle (3-HP), 3-Hydroxypropionate/4-hydroxybutylate cycle (HP-HB), and Dicarboxylate/4-hydroxybutyrate cycle (DC-HB), and the functional genes that were selected were detected in the results of the assay with high relative abundance (Evans et al., 1966; Ljungdhal, 1986; Holo, 1989; Berg et al., 2007; Huber et al., 2008; Frolov et al., 2019). Nitrogen cycle-related functional genes were selected based on the important pathways of the nitrogen cycle that could be detected in the test results with high relative abundance (Canfield et al., 2010; Kuypers et al., 2018). Relative abundances of functional categories and taxa were averaged for each treatment, and visualized as heat maps using the Origin 2021. The correlation between microbial taxa and soil properties was measured by Spearman's rank correlation coefficient and visualized using the R package “ggplot.”

## 3 Results

### 3.1 Effects of N deposition on soil physical-chemical properties and plant biomass

AGB and BGB tended to increase with increasing N deposition but did not reach significant levels (Table 1). At the N50 treatment, NO${\;}_{3}^{-}$-N, NH${\;}_{4}^{+}$-N, and DOC content significantly increased after N addition (*P* < 0.05, Table 1). However, at the N30 treatment, no differences were detected between the N addition and control plots. No other soil properties changed significantly in response to the N30 and N50 treatment (Table 1). Soil pH and TN decreased with the increase in N concentration, while SOC increased with the increase of N concentration (Table 1).

**Table 1:** Effects of N deposition on plant biomass and soil physicochemical properties in desert steppe.

**Variable**	**N0**	**N30**	**N50**
AGB (g m^−2^)	153.95a	157.41a	159.53a
BGB (g m^−2^)	1,612.01a	1,702.3a	1,842.09a
pH	8.38a	8.36a	8.30a
SOC (g kg^−1^)	18.84a	20.64a	20.91a
DOC (mg kg^−1^)	83.71b	92.10ab	100.75a
TN (g kg^−1^)	1.88a	1.84a	1.83a
NO${\;}_{3}^{-\text{\_}}$N (mg kg^−1^)	9.46b	12.72b	28.89a
NH${\;}_{4}^{+\text{\_}}$N (mg kg^−1^)	1.38b	2.36b	5.93a

Values are means (n = 4).

AGB, above-ground biomass; BGB, below-ground biomass; SOC, soil organic C; DOC, soil dissolved soil organic C; TN, total N; N0, control; N30, N addition 30 kg ha^−1^ year^−1^; N50, N addition 50 kg ha^−1^ year^−1^.

Lowercase letters indicate differences among the three N treatments (*P* < 0.05).

### 3.2 Effects of N deposition on microbial taxa and functions

**Figure 1:**
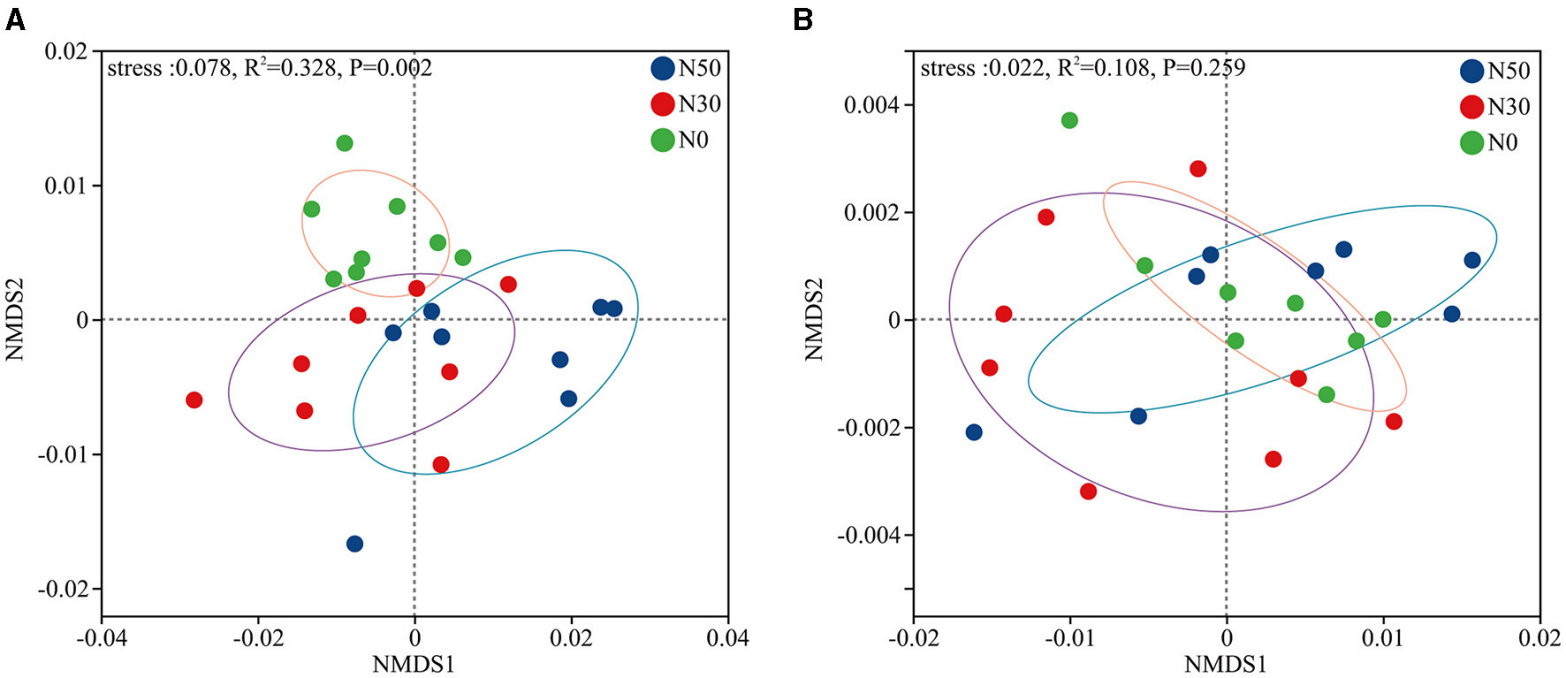
According to the NMDS analysis based on the principles of **(A)** Phyla and **(B)** Kyoto Encyclopedia of Genes and Genomes (KEGG, level-3 paths), N deposition affects microbial β-diversity. N0, control; N30, N addition 30 kg ha^−1^ year^−1^; N50, N addition 50 kg ha^−1^ year^−1^. Four replications, with 2 samples per repetition.

PERMANOVA analysis showed that N deposition significantly affected the microbial β-diversity of desert steppe (Figure 1A, *P* < 0.05). However, N deposition did not significantly affect the β-diversity of functional genes (KEGG, level-3 paths; Figure 1B).

Compared with N0 treatments, the relative abundance of *Actinobacteria, Firmicutes, Uroviricota*, Candidatus *Firestonebacteria*, Candidatus *Komeilibacteria*, and *Kitrinoviricota* increased significantly in N50 treatment (Table 2, *P* < 0.05). Compared with the N0 treatment, the relative abundance of *Proteobacteria, Acidobacteria, Bacteroidetes, Abditibacteriota*, and *Discosea* in the N50 treatment decreased significantly (Table 2, *P* < 0.05). Compared with the N30 treatment, the relative abundance of *Ascomycota, Chlamydiae, Apicomplexa*, and *Candidatus Brennerbacteria* in the N50 treatment decreased significantly (Table 2, *P* < 0.05). The relative abundance of *Thaumarchaeota*, Candidatus *Bathyarchaeota, Ascomycota, Basidiomycota, Chlamydiae, Apicomplexa*, and Candidatus *Brennerbacteria* showed an increasing trend followed by a decreasing trend with increasing N deposition, with the highest relative abundance observed in the N30 treatment (Table 2).

**Table 2:** Effects of N deposition on the relative abundance of major microorganisms.

**Microbial taxa**	**N0**	**N30**	**N50**
*Actinobacteria*	61.84b	61.91b	63.44a
*Proteobacteria*	14.81a	14.20a	13.51b
*Acidobacteria*	4.84a	4.66ab	4.30b
*Thaumarchaeota*	4.14b	4.59a	4.32ab
*Firmicutes*	4.61E-01b	4.67E-01ab	4.81E-01a
*Bacteroidetes*	2.49E-01a	2.50E-01a	2.26E-01b
Candidatus *Bathyarchaeota*	1.66E-02b	1.80E-02a	1.76E-02ab
*Ascomycota*	8.63E-03ab	9.24E-03a	6.36E-03b
*Abditibacteriota*	6.98E-03a	7.00E-03a	6.41E-03b
*Uroviricota*	5.27E-03b	5.93E-03ab	7.17E-03a
*Basidiomycota*	4.03E-03b	5.19E-03a	4.34E-03ab
*Chlamydiae*	3.92E-03ab	4.20E-03a	3.37E-03b
*Apicomplexa*	1.08E-03ab	1.21E-03a	9.20E-05b
Candidatus *Komeilibacteria*	2.31E-04b	2.43E-04b	3.34E-04a
Candidatus *Firestonebacteria*	1.23E-04b	1.28E-04ab	1.82E-04a
*Kitrinoviricota*	3.34E-05b	8.43E-05b	1.73E-04a
*Discosea*	8.08E-05a	2.65E-05b	3.41E-05b
Candidatus *Brennerbacteria*	1.75E-05ab	4.32E-05a	9.62E-06b

Value represents the means relative abundance (n = 4).

N0, control; N30, N addition 30 kg ha^−1^ year^−1^; N50, N addition 50 kg ha^−1^ year^−1^.

Lowercase letters indicate differences among the three N treatments (*P* < 0.05).

**Figure 2:**
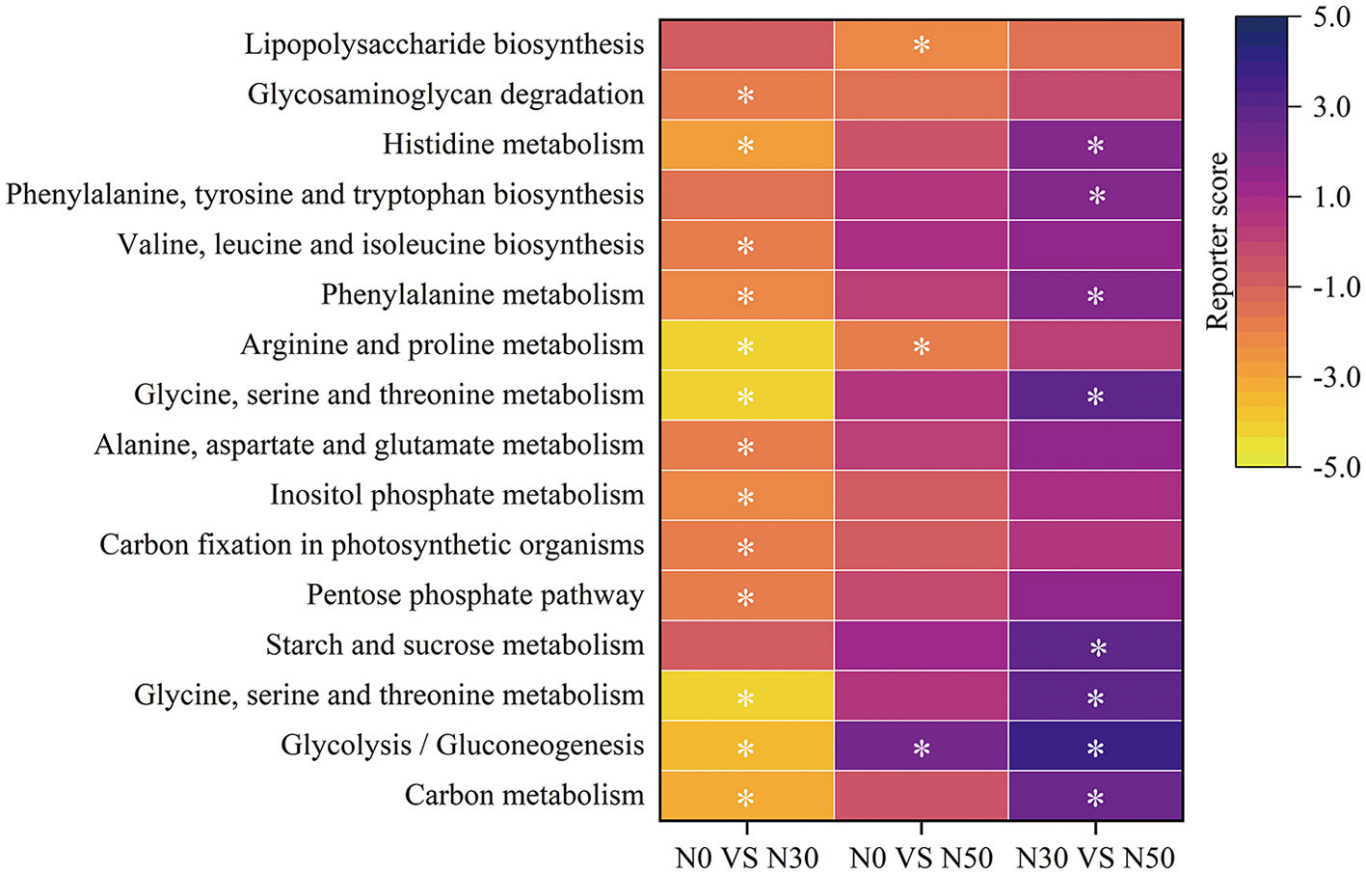
KEGG pathway level-3 enrichment analysis. Yellow, enriched in control; purple, enriched in treatment. N0 vs. N30, N0 was the control group and N30 was the treatment group; N0 vs. N50, N0 was the control group and N50 was the treatment group; N30 vs. N50, N30 was the control group and N50 was the treatment group. Asterisk denotes reporter score of pathways >1.65 or < -1.65. Functions significantly enriched for reporter score absolute values ≥1.65.

Enrichment analysis screened 16 metabolic pathways related to C and N cycling, which were significantly changed after N treatment (Figure 2). Genes of C metabolism, Glycolysis/Gluconeogenesis, Glycine, serine and threonine metabolism, Pentose phosphate pathway, C fixation in photosynthetic organisms, Inositol phosphate metabolism, Alanine, aspartate and glutamate metabolism, Glycine, serine and threonine metabolism, Arginine and proline metabolism, Phenylalanine metabolism, Valine, leucine and isoleucine biosynthesis, Histidine metabolism, and Glycosaminoglycan degradation metabolic pathways were significantly enriched in N0 compared to N30 treatment (Figure 2; Reporter score < -1.65; Functions significantly enriched for reporter score absolute values ≥1.65). Genes of Arginine and proline metabolism and Lipopolysaccharide biosynthesis metabolic pathways were significantly enriched in the N0 treatment compared to the N50 treatment (Figure 2; Reporter score < -1.65). Glycolysis/Gluconeogenesis metabolic pathway genes were significantly enriched in the N50 treatment compared to the N0 treatment (Figure 2; Reporter score >1.65). Genes of C metabolism, Glycolysis/Gluconeogenesis, Glycine, serine and threonine metabolism, Starch and sucrose metabolism, Glycine, serine and threonine metabolism, Phenylalanine metabolism, Phenylalanine, tyrosine and tryptophan biosynthesis, and Histidine metabolism metabolic pathways were significantly enriched in N50 compared to N30 treatment (Figure 2, Reporter score >1.65). Among them, compared to the N0 and N30 treatments, the N50 treatment significantly increased the relative abundance of the Glycolysis/Gluconeogenesis module (Figure 2; Reporter score >1.65).

**Figure 3:**
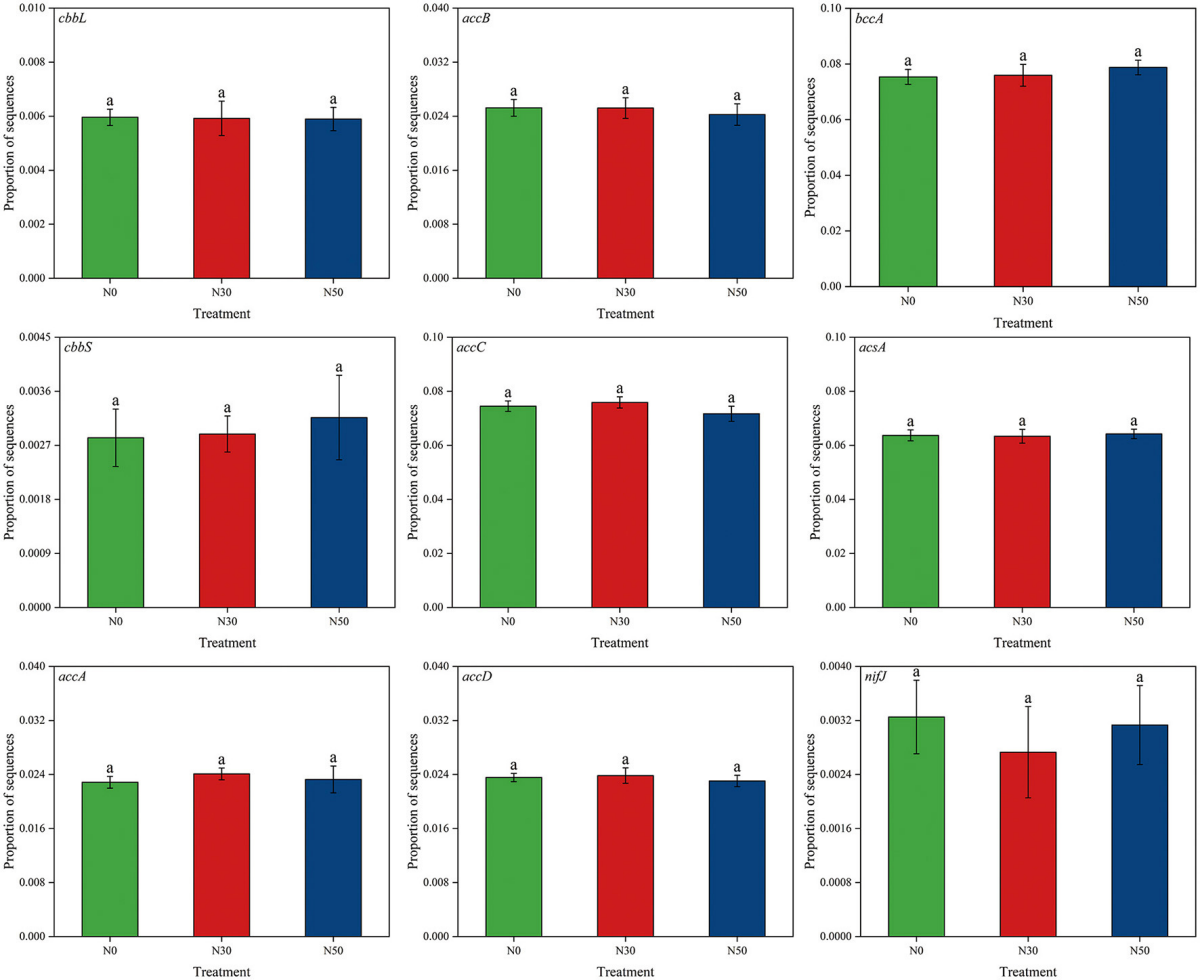
Effects of N deposition on the abundance of functional genes related to the C cycle in a desert steppe. N0, control; N30, N addition 30 kg ha^−1^ year^−1^; N50, N addition 50 kg ha^−1^ year^−1^. Lowercase letters indicate differences among the three N treatments (*P* < 0.05).

Compared with N0, N deposition (N30 and N50) did not significantly affect the abundance of functional genes related to the C cycle (*cbbL, cbbS, accA, accB, accC, accD, bccA, acsA, nifJ*; *P* < 0.05, Figure 3).

**Figure 4:**
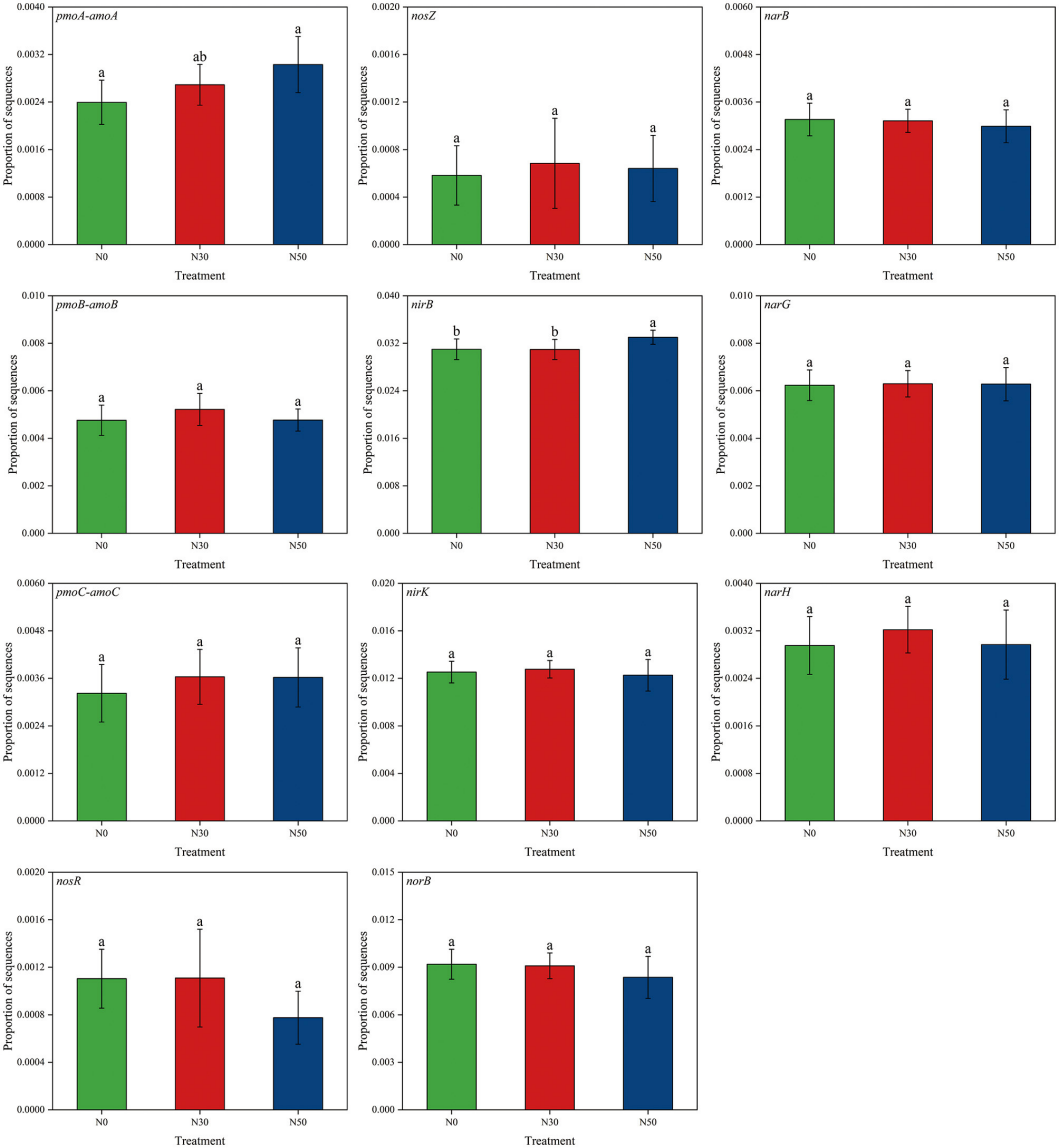
Effects of N deposition on the abundance of functional genes related to the N cycle in a desert steppe. N0, control; N30, N addition 30 kg ha^−1^ year^−1^; N50, N addition 50 kg ha^−1^ year^−1^. Lowercase letters indicate differences among the three N treatments (*P* < 0.05).

Compared to N0, the abundance of *pmoA-amoA* and *nirB* genes was significantly increased in the N50 treatment (*P* < 0.05), while there was no significant difference observed in the N30 treatment (Figure 4). However, there was no significant difference in other genes related to the N cycle (*pmoB-amoB, pmoC-amoC, nosR, nosZ, nirK, norB, narB, narG, narH*; *P* < 0.05, Figure 4). Compared with N0, *nosR, nirK, norB*, and *narB* gene showed a downward trend in N50 treatment (Figure 4).

### 3.3 Correlation between C cycling-related microorganisms and environmental variables

**Figure 5:**
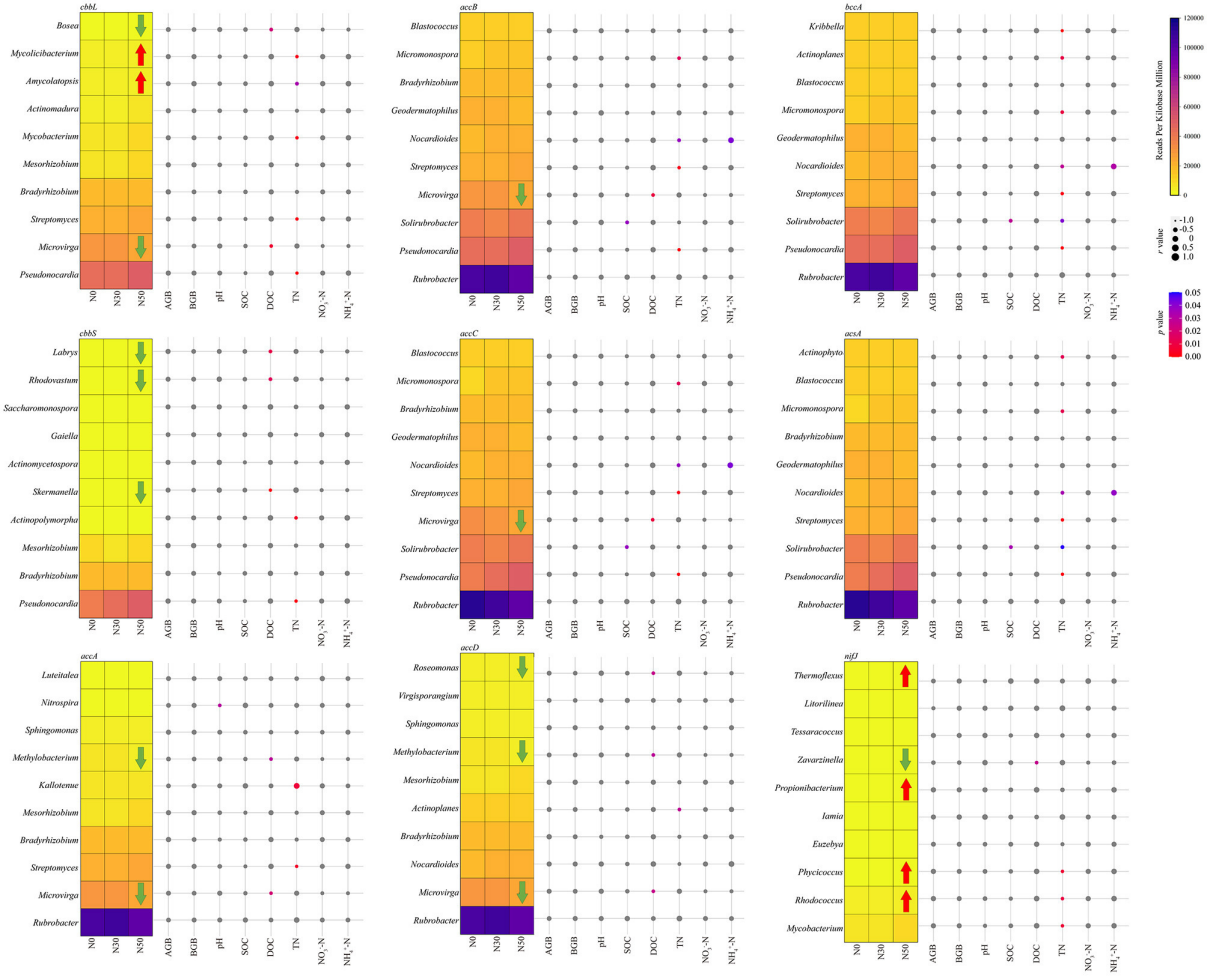
Heatmap shows the abundance changes of the dominant genera. Taxonomic classification was performed using C cycle functional genes. The red arrow indicates that the relative abundance is significantly increased compared with the control (N0). In contrast, the green arrow indicates that the relative abundance is significantly lower than that of the control (N0). The bubble chart displays the Spearman correlation coefficients between dominance and environmental variables. Correlation analysis was conducted based on the abundance of taxa containing each C cycle functional gene. AGB, aboveground biomass; BGB, belowground biomass; SOC, soil organic C; DOC, dissolved organic C; TN, total N; NO${\;}_{3}^{-}$-N, nitrate N; NH${\;}_{4}^{+}$-N, ammonium N.

*Rubrobacter* carries *accA, accB, accC, accD, bccA, and acsA* genes, and its abundance is the highest in the N30 treatment. It is also the highest relative abundance among microorganisms involved in the C cycle (Figure 5). The relative abundance of *Pseudonocardia* carrying *cbbL, cbbS, accB, accC, bccA*, and *acsA* increased with N deposition (Figure 5). *Microvirga* carried *cbbL, accA, accB, accC*, and *accD* genes, and its relative abundance decreased significantly compared with N0 in N50 treatment (*P* < 0.05, Figure 5). *Methylobacterium* carried *accA* and *accD* genes, and its relative abundance decreased significantly compared with N0 in N50 treatment (*P* < 0.05, Figure 5). *Microvirga* and *Bosea* carrying cbbL gene's relative abundance decreased significantly, while *Amycolatopsis* and *Mycolicibacterium*'s relative abundance increased significantly in N50 treatments (*P* < 0.05, Figure 5). The relative abundance of *Skermanella, Rhodovastum*, and *Labrys* carrying *the cbbS* gene decreased significantly in the N50 treatment (*P* < 0.05, Figure 5). The relative abundance of *Zavarzinella* carrying the *nifJ* gene decreased significantly, while the relative abundance of *Rhodococcus, Phycicoccus, Propionibacterium*, and *Thermoflexus* increased significantly in N50 treatments (*P* < 0.05, Figure 5).

The correlation between AGB and BGB and C cycling-related microorganisms is insignificant (Figure 5). *Nitrospira* was significantly negatively correlated with pH (*P* < 0.05, Figure 5). *Solirubrobacter* was significantly negatively correlated with SOC (*P* < 0.05, Figure 5). DOC has a significant negative correlation with eight genera of microorganisms: *Microvirga, Methylobacterium, Roseomonas, Bosea, Skermanella, Rhodovastum, Labrys*, and *Zavarzinella* (*P* < 0.05, Figure 5). TN was significantly negatively correlated with 13 genera of microorganisms, including *Pseudonocardia* (*P* < 0.05, Figure 5). NO${\;}_{3}^{-}$-N was not significantly correlated with C cycle-related microorganisms (Figure 5). *Nocardioides* were significantly positively correlated with NH${\;}_{4}^{+}$-N (*P* < 0.05, Figure 5).

### 3.4 Correlation between N cycling-related microorganisms and environmental variables

**Figure 6:**
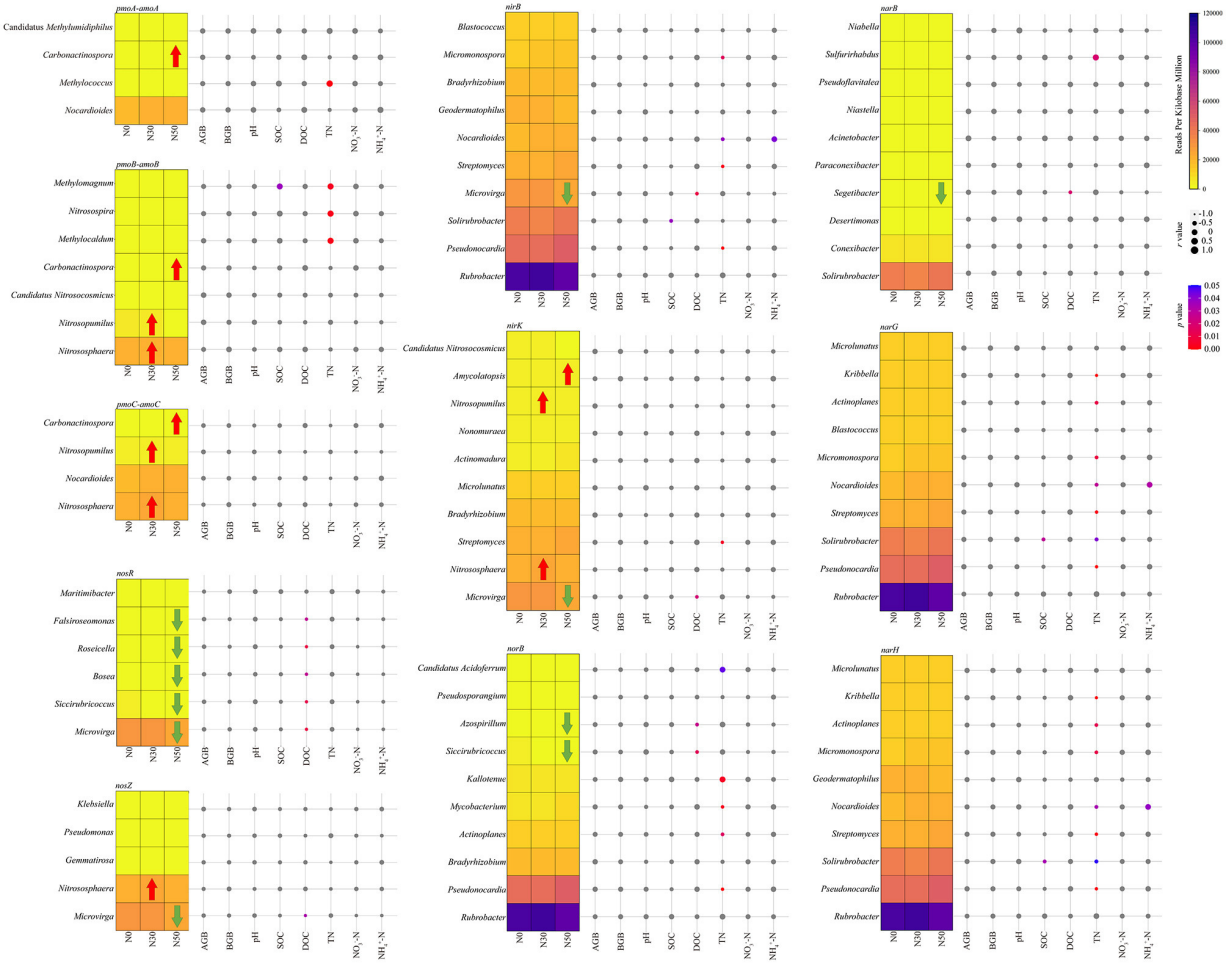
Heatmap shows the abundance changes of the dominant genera. Taxonomic classification was performed using N cycle functional genes. The red arrow indicates that the relative abundance is significantly increased compared with the control (N0). In contrast, the green arrow indicates that the relative abundance is significantly lower than that of the control (N0). The bubble chart displays the Spearman correlation coefficients between dominance and environmental variables. Correlation analysis was conducted based on the abundance of taxa containing each N cycle functional gene. AGB, aboveground biomass; BGB, belowground biomass; SOC, soil organic C; DOC, dissolved organic C; TN, total N; NO${\;}_{3}^{--}$N, nitrate N; NH${\;}_{4}^{+}$-N, ammonium N.

*Rubrobacter* and *Pseudonocardia* are important microorganisms in the C cycle and the N cycle, and they both carry *nirB, norB, narG*, and *narH* genes (Figure 6). *Microvirga* carried *nosR, nosZ, nirB*, and *nirK* genes (Figure 6). *Nitrososphaera* carried *pmoB-amoB, pmoC-amoC, nosZ*, and *nirK* genes, and its relative abundance increased significantly compared with N0 in N30 treatment (*P* < 0.05, Figure 6). *Carbonactinospora* carried *pmoA-amoA, pmoB-amoB*, and *pmoC-amoC* genes, and its relative abundance increased significantly compared with N0 in N50 treatment (*P* < 0.05, Figure 6). The significant increase in the *pmoA-amoA* gene in N50 treatment is also related to the increase in the relative abundance of *Carbonactinospora* (*P* < 0.05, Figure 6). *Nitrosopumilus* carried *pmoB-amoB* and *pmoC-amoC* genes, and its relative abundance increased significantly compared with N0 in N50 treatment (*P* < 0.05, Figure 6). The relative abundance of *Microvirga, Siccirubricoccus, Bosea, Roseicella*, and *Falsiroseomonas* carrying the *nosR* gene decreased significantly in N50 treatment (*P* < 0.05, Figure 6). The relative abundance of *Siccirubricoccus* and *Azospirillum* carrying *norB* gene decreased significantly in N50 treatment (*P* < 0.05, Figure 6). The relative abundance of *Segetibacter* carrying the *narB* gene significantly reduced in the N50 treatment (*P* < 0.05, Figure 6).

AGB, BGB, and NO${\;}_{3}^{-}$-N was likewise not significantly correlated to N-cycling microorganisms (Figure 6). Soil pH was not significantly correlated with N cycle-related microorganisms (Figure 6). *Methylomagnum* positively correlated with SOC (*P* < 0.05, Figure 5). DOC has a significant negative correlation with seven genera of microorganisms: *Microvirga, Siccirubricoccus, Roseicella, Bosea, Falsiroseomonas, Azospirillum*, and *Segetibacter* (*P* < 0.05, Figure 6).TN was significantly negatively correlated with eight genera of microorganisms, including *Actinoplanes, Kribbella, Micromonospora, Mycobacterium, Nocardioides, Pseudonocardia, Solirubrobacter*, and *Streptomyces* (*P* < 0.05, Figure 6). TN was significantly positively correlated with seven genera of microorganisms, including *Candidatus Acidoferrum, Kallotenue, Methylocaldum, Methylococcus, Methylomagnum, Nitrosospira*, and *Sulfurirhabdus* (*P* < 0.05, Figure 6). SOC and NH${\;}_{4}^{+}$-N were consistent with C cycle microbial results.

## 4 Discussion

### 4.1 Effect of N deposition on the composition of soil microbial community

Several data suggest that N deposition significantly affects soil microbial community composition. Compared to N0, the relative abundance of *Actinobacteria* was increased dramatically in the N50 treatment, while there was no significant change in the N30 treatment. *Actinobacteria* were the microorganisms with the highest relative abundance in desert steppe. Most *Actinobacteria* were saprophytic heterotrophic bacteria that can utilize C sources in the soil, and N sources such as nitrate and ammonium N can also be used by them (Fierer et al., 2007; Araujo et al., 2020). Nitrogen deposition increases AGB and BGB in a desert steppe. This increase in biomass may provide a C source for the growth of *Actinobacteria*, which could be one of the main reasons for their relative abundance increase (Dai et al., 2018). A study of global grassland microbial communities showed that the relative abundance of copiotrophic soil bacterial groups such as *Actinobacteria* increased with N nutrient addition, confirming the results of this study (Leff et al., 2015).

Compared to N0, the relative abundance of *Proteobacteria* was significantly decreased in the N50 treatment. Most microorganisms in *Proteobacteria* are known as copiotrophic life history strategies, and it is generally believed that the relative abundance of *Proteobacteria* increases with increasing N deposition (Noah et al., 2012; Grant et al., 2022; Ramotowski and Shi, 2022). Especially in the case of *Alphaproteobacteria*, its response to N addition is typically positive. However, in this study, *Alphaproteobacteria* was the most abundant group within *Proteobacteria*, and the decline reduction in the relative abundance of *Alphaproteobacteria* caused the decline in the relative abundance of *Proteobacteria*. This inconsistency may be because bacteria are highly environment-dependent or selectively evolutionarily conserved in their response to N (Isobe et al., 2019). In another study conducted on a semiarid steppe, similar findings were observed where the relative abundance of *Alphaproteobacteria* showed a decreasing trend with N addition. However, it did not reach statistical significance (Liu et al., 2020).

Compared to N0, the relative abundance of *Acidobacteria* was significantly decreased in the N50 treatment. *Acidobacteria* can adapt to soils with relatively low pH. Nitrogen deposition decreases the soil pH in the desert steppe, but the relative abundance of *Acidobacteria* does not increase with the decrease in pH (Wang et al., 2018). A global study has indicated that *Acidobacteria* is an oligotrophic bacteria, and N deposition inhibits its development rather than increasing with decreasing pH (Dai et al., 2018).

*Ascomycota* and *Basidiomycota* were the fungi with the highest relative abundance, and their relative abundance was the highest in the N30 treatment. Compared with the N30 treatment, the relative abundance of *Ascomycota* decreased significantly in the N50 treatment. Nitrogen deposition has different effects on the relative abundance of *Ascomycota* and *Basidiomycota*, which have both positive and negative effects (Weber et al., 2013; Wang et al., 2021; Taylor Andy et al., 2022). This is mainly due to fungi's high N conservative, meaning that they do not necessarily require large amounts of N to meet their growth needs (Allison et al., 2009). Therefore, the N addition rate does not have a linear relationship with the relative abundance of *Ascomycota* and *Basidiomycota* in this study.

### 4.2 The effect of N deposition on microbial functional genes for soil C-N cycling

The annual addition or deposition of 50 kg ha^−1^ year^−1^ for up to 6 years did not affect the C cycle gene abundance but changed the C cycle-related microorganism community structure. The microorganisms *Rubrobacter, Pseudonocardia*, and *Microvirga* play a crucial role in the C-cycling process in the desert steppe, often harboring various C-cycling functional genes. However, protein-coding genes typically undergo highly frequent horizontal gene transfer, which may lead to false positive results. *Rubrobacter* and *Pseudonocardia* both belong to the *Actinobacteria*. They are closely related to the C cycle (including C fixation and metabolism), and their relative abundance is relatively high, which increases with N deposition (Huang et al., 2022). *Microvirga*, which belongs to the *Proteobacteria*, is a major contributor to the Calvin cycle, and its relative abundance decreases progressively with increasing nitrogen deposition (Huang et al., 2022). It was replaced by *Rubrobacter*, an important participant in the Calvin cycle (Huang et al., 2022). Several microorganisms (*Amycolatopsis, Propionibacterium, Rhodococcus, Phycicoccus*, and *Thermoflexus*) of relatively low abundance increased with N addition. Meanwhile, *Bosea, Labrys, Rhodovastum, Skermanella, Methylobacterium, Roseomonas*, and *Zavarzinella* decreased with the N addition. Among them, most microorganisms (such as: *Pseudonocardia* and *Amycolatopsis*) showed a significant negative correlation with TN, which may be due to the increase in gross N transformations in alpine calcareous soil caused by N application, leading to increased N availability and subsequently promoting the growth of these microorganisms (Hao et al., 2020). The relative abundance of most microbial genera involved in the same C cycle process is correlated with TN, indicating niche overlap for C cycle taxa (Michalska-Smith et al., 2022). Nitrogen deposition leads to a decrease in the relative abundance of one type of C-cycling microorganisms while at the same time contributing to an increase in the abundance of a different kind of microorganisms with the same function, which may be another major reason why N deposition does not significantly reduce or increase the C-cycling function genes of the desert steppe.

Furthermore, *Rubrobacter, Pseudonocardia*, and *Microvirga* play crucial roles not only in C cycling but also in N cycling. In this study, N deposition (50 kg ha^−1^ year^−1^) significantly increased the relative abundance of *pmoA-amoA* and *nirB* genes. The *pmoA-amoA* is considered to be related to nitrification, and the increase in its abundance represents the enhancement of nitrification (Wang et al., 2023b). The study reported that autotrophic nitrification was mainly stimulated when N deposition was below 55 kg ha^−1^ year^−1^, thus contributing to the stimulation of nitrification (Song et al., 2021). This is mainly because autotrophic nitrifiers acquire energy from the process of ammoxidation. At the same time, the increase in soil NH${\;}_{4}^{+}$ also favors autotrophic nitrification, a conclusion that can be verified by the significant rise in NH${\;}_{4}^{+}$-N content in this study (Wrage et al., 2001; Song et al., 2021). The *nirB* gene produces an assimilatory nitrite reductase that catalyzes the assimilatory uptake of NO${\;}_{2}^{--}$ for reduction to NH${\;}_{4}^{+}$ (Kuypers et al., 2018). This could also be another major reason for the increase in soil NH${\;}_{4}^{+}$-N content, which is the main reason for the significant increase in both *pmoA-amoA* and *nirB* genes. Increased relative abundance of *Nocardioides* is one of the important factors in the increased abundance of *pmoA-amoA* and *nirB* genes. *Nocardioides* belong to *Actinobacteria*, known as inert communities, which can form endospores to resist environmental changes under drought stress (Li et al., 2022a). Increased relative abundance of *Streptomyce* is also an important reason for the increase in the *nirB* gene. *Actinobacteria* can secrete antibiotics and biosynthesize compounds such as geosmin and 2-MIB, and their production also contributes to the spread of *Streptomyce* in the soil using *Springtail* (Bei et al., 2023). *Nitrososphaera* belongs to *Thaumarchaeota* and carries *pmoB-amoB, pmoC-amoC, nosZ*, and *nirK* genes, important microorganisms in the N cycle process. Both increased their relative abundance with N deposition, a result that is consistent with previous studies (Bei et al., 2023). *Nitrosopumilus* also belongs to *Thaumarchaeota* and, in this study, mainly carries the *pmoB-amoB, pmoC-amoC*, and *nirK* genes, which also proved to be an important participant in the N cycle of the desert steppe (Wang et al., 2023b). There may also be niche overlap for microorganisms involved in N cycling processes.

## 5 Conclusion

In conclusion, our study emphasizes the effects of 6 years of N deposition on soil microbial community composition and C and N cycling functions in desert steppe. This study suggests that N deposition significantly affected the composition of soil microbial community in desert steppe; Annual addition of 50 kg ha^−1^ year^−1^ for up to 6 years did not affect the C cycle gene abundance but changed the C cycle-related microorganism community structure; The process of the N cycle in desert steppe was affected by N deposition, which increased the abundance of *pmoA-amoA* gene related to nitrification and *nirB* gene related to assimilation nitrite reductase. Ecologically niche overlap between microorganisms involved in the same C and N cycling processes may exist. The research findings are crucial for assessing how N deposition affects the C and N function in arid and semi-arid grassland ecosystems, and gaining a better understanding of the ecological consequences of anthropogenic N addition.

## Data availability statement

The datasets presented in this study can be found in online repositories. The names of the repository/repositories and accession number(s) can be found in the article/Supplementary material.

## Author contributions

HY: Data curation, Formal analysis, Funding acquisition, Writing – original draft. YZ: Formal analysis, Writing – original draft. SH: Formal analysis, Writing – original draft. ZW: Formal analysis, Writing – original draft. MY: Data curation, Writing – original draft. MH: Data curation, Formal analysis, Funding acquisition, Supervision, Writing – review & editing.
