# Supplementary material for: Metagenomics reveals the response of desert steppe microbial communities and carbon-nitrogen cycling functional genes to nitrogen deposition
Source: Front Microbiol. 2024 Mar 26;15:1369196. doi: 10.3389/fmicb.2024.1369196 (PMC11002186; doi:10.3389/fmicb.2024.1369196)
Supplement: Supplementary file 1 [file Table_1.docx]

Supplementary Material

## Supplementary Tables

**Supplementary Table 1. The amount of NH_4_NO_3_ used in each treatment (g)**

| Month | N0 | N30 | N50 |
| --- | --- | --- | --- |
| January | 0.00 | 2.21 | 3.68 |
| February | 0.00 | 5.14 | 8.56 |
| March | 0.00 | 4.94 | 8.23 |
| April | 0.00 | 12.32 | 20.53 |
| May | 0.00 | 27.82 | 46.36 |
| June | 0.00 | 79.86 | 133.11 |
| July | 0.00 | 119.51 | 199.18 |
| August | 0.00 | 57.07 | 95.12 |
| September | 0.00 | 67.06 | 111.77 |
| October | 0.00 | 30.23 | 50.38 |
| November | 0.00 | 25.35 | 42.25 |
| December | 0.00 | 6.00 | 10.00 |
| Total | 0.00 | 437.51 | 729.17 |

Note：N0, control; N30, N addition 30 kg ha^-1^ yr^-1^; N50, N addition 50 kg ha^-1^ yr^-1^. Each plot has an area of 49 m^2^.
